# The last marine pelomedusoids (*Testudines*: *Pleurodira*): a new species of *Bairdemys* and the paleoecology of *Stereogenyina*

**DOI:** 10.7717/peerj.1063

**Published:** 2015-06-30

**Authors:** Gabriel S. Ferreira, Ascanio D. Rincón, Andrés Solórzano, Max C. Langer

**Affiliations:** 1 Laboratório de Paleontologia de Ribeirão Preto, FFCLRP, Universidade de São Paulo, Ribeirão Preto, SP, Brazil; 2 Laboratorio de Paleontología, Centro de Ecología, Instituto Venezolano de Investigaciones Científcas (IVIC), Caracas, Venezuela

**Keywords:** Bairdemys, Stereogenyina, Podocnemididae, Marine pelomedusoides, Paleoecology, Durophagy, Miocene, Venezuela

## Abstract

The extinct *Stereogenyina* turtles form a relatively diverse *Podocnemididae* lineage, with twelve described and phylogenetically positioned species. They are characterized by a wide geographic and temporal range, from the Eocene of Africa to the Pleistocene of Southeast Asia, and a peculiar palate morphology, with a secondary palate that is unique among side-necked turtles. Here, we describe a new *Stereogenyina* species, based on an almost complete skull from the middle Miocene Capadare Formation, of Venezuela. A new phylogenetic analysis supports the assignment of the new species to the genus *Bairdemys*. Based on geometric morphometrics analyses, we related the development of the stereogenyin secondary palate with the acquisition of a durophagous diet. Based on a review of the sedimentary environments where their fossils are found, we also propose that stereogenyins were a marine radiation of podocnemidid turtles, as corroborated by previous studies of fossil eggs and limb morphology. These two inferences allowed us to hypothesize that stereogenyins occupied an ecological niche similar to that of the extant Carettini sea turtles, and that the rise of the latter group may be related to the *Stereogenyina* diversity fall in the end of the Miocene.

## Introduction

The *Stereogenyina* is a peculiar extinct podocnemidid lineage, promptly recognized among pleurodiran turtles by their unique secondary palate, with a midline cleft formed by the maxilla and the palatine (Gaffney et al., 2011). With a relatively rich fossil record, the oldest known stereogenyins are *Cordichelys antiqua* (Andrews, 1903) and *Stereogenys cromeri* (Andrews, 1901) from the late Eocene of the Birket el-Qurun Formation, in Egypt, and its most recent record is *Shweboemys pilgrimi* (Swinton, 1939) from the Plio-Pleistocene Irrawaddy Formation, in Burma. The broad geographical range of the group includes North and South America, North Africa, and Southeast Asia (Gaffney et al., 2011).

Six of the twelve known stereogenyin species have been formally assigned to *Bairdemys* (Weems & Knight, 2013), a genus known from the Oligocene of the USA to the Miocene of Puerto Rico and Venezuela. They are characterized by the curved edges of the secondary palate midline cleft, and the very small, slitlike opening of the antrum postoticum (Gaffney et al., 2008). The first known taxon of this group was described by Wood & Díaz de Gamero (1971) as *Podocnemis venezuelensis*, from the late Miocene Urumaco Formation, Venezuela. It was inferred as possibly corresponding to a marine turtle, on the basis of the depositional environment of the type locality (Wood & Díaz de Gamero, 1971), as supported by further evidence (Winkler & Sánchez-Villagra, 2006; Smith et al., 2010).

**Figure 1:**
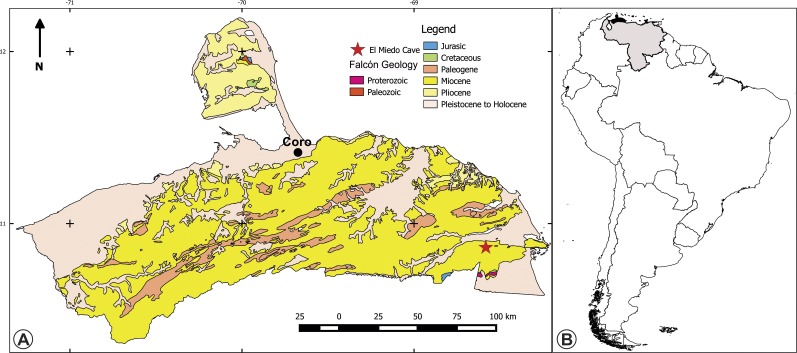
Maps of the type location of the holotype of *Bairdemys thalassica*. (A) Surface geology map of the Falcón state, Venezuela, showing the location of the El Miedo Cave, where the holotype of *Bairdemys thalassica* was found, and (B) map of South America showing Venezuela (light gray) and Falcón state (black).

Here we describe a new species of *Bairdemys*, from the marine carbonatic deposits of the middle Miocene Capadare Formation, Venezuela (Fig. 1). We further explore the structure of the secondary palate of *Stereogenyina* and evidences of their marine adaptations, allowing a better understanding of the evolutionary history of these unique pleurodiran turtles.

## Materials & Methods

The fossil material was found partially encased into a limestone bed, and was prepared using acid solution. The material was described comparatively to other pelomedusoids, following the anatomical nomenclature used by Gaffney (1979), Gaffney, Tong & Meylan (2006) and Gaffney et al. (2011).

### Nomenclatural acts

The electronic version of this article in Portable Document Format (PDF) will represent a published work according to the International Commission on Zoological Nomenclature (ICZN), so that the new names contained in the electronic version are effectively published under that Code from the electronic edition alone. This published work and the nomenclatural acts it contains have been registered in ZooBank, the online registration system for the ICZN. The ZooBank LSIDs (Life Science Identifiers) can be resolved and the associated information viewed through any standard web browser by appending the LSID to the prefix “http://zoobank.org/”. The LSID for this publication is: [urn:lsid:zoobank.org:pub:F431F398-5442-4917-83E7-04CD0598225A]. The online version of this work is archived and available from the following digital repositories: PeerJ, PubMed Central and CLOCKSS.

Clade names follow, whenever possible, Phylogenetic Nomenclature principles and rules, as defined by the PhyloCode v. 4.0 (Cantino & de Queiroz, 2010). As such, suprageneric italicized names have been phylogenetically defined and those non-italicized refer to names defined in the context of the International Code of Zoological Nomenclature (ICZN, 1999).

The newly described skull was coded into a taxon-character matrix using Mesquite v. 3.0 (Maddison & Maddison, 2014). The matrix includes 57 characters (nine of which are new and 48 previously proposed by various authors) (SI1; Bona & de la Fuente, 2005; Cadena et al., 2012; Dumont Júnior, 2013; de la Fuente, 2003; Gaffney, 1977; Gaffney, Tong & Meylan, 2006; Gaffney et al., 2011; Meylan, Gaffney & Campos, 2009; Thomson & Georges, 2009, and 16 taxa. *Podocnemis unifilis* (Troschel, 1848), *Peltocephalus dumerilianus* (Schweigger, 1812), *Erymnochelys madagascariensis* (Grandidier, 1867) and *Mogharemys blackenhorni* (Dacqué, 1912) formed the outgroup and twelve *Stereogenyina* were included in the ingroup. The resulting matrix was analysed in TNT v. 1.0 (Goloboff, Farris & Nixon, 2008) using parsimony and implicit enumeration algorithms and bootstrap values (GC, 1,000 replicates, Fig. 5; Goloboff et al., 2003) were calculated using TNT implemented functions. A second analysis was run in TNT with a positive constraint (using the command *force*) for the monophyly of all species previously assigned to *Bairdemys*, and the resulting suboptimal topology was compared with the most parsimonious tree (MPT) using the Templeton test (Templeton, 1983) in PAUP* v. 4.0 (Swofford, 2002) (Table 1).

**Table 1:** Summary of the results of the Templeton test. Output of the Templeton test of the most parsimonious tree and one suboptimal tree with positive constraint for the monophyly of *Bairdemys*.

Templeton (Wilcoxon signed-ranks) test:	
*N*	11
Test statistic (*T*)	0
*P*	0.0021^∗^

Two geometric morphometric analyses were performed to test if the morphology of the triturating surface of the upper and lower jaws of *Stereogenyina* corresponds to that of a durophagous turtle (Claude et al., 2004). The morphometric analysis of the upper jaw employed 14 extant taxa and three *Stereogenyina* with well preserved upper jaws. The lower jaw analysis employed 15 extant taxa plus three *Stereogenyina* with well preserved mandibles. The extant taxa used in both analyses were chosen based on the knowledge of their feeding habits (Moll , 1976; Meylan, 1988; Burke, Morreale & Standora, 1994; Teran, Vogt & Gomez, 1995; Pérez-Emán & Paolillo, 1997; Claude et al., 2004; Polovina et al., 2004; Salmon, Jones & Horch, 2004; Garcia & Lourenco, 2007). The list of employed specimens is seen in Supplemental Information 3. The skulls were photographed in ventral view and the lower jaws in dorsal view, and landmarks were digitized using the software tpsDig2 (Rohlf, 2005). Twelve landmarks for the upper and ten for the lower jaw were chosen (Fig. 6; description of the landmarks on Supplemental Information 3), aiming at representing the size and general shape of those structures. The shape data were extracted from the landmark dataset by a Generalized Procrustes Analysis (GPA, Rohlf, 1999) in the software MorphoJ v. 1.06d (Klingenberg, 2011), taking into account object symmetry (Klingenberg & McIntyre, 1998; Klingenberg, Barluenga & Meyer, 2002). Principal Component Analysis (PCA) (Table 2; Figs. 7A and 7C) were conducted for each dataset and components that explained morphological variation were retained after analysis. We tested the normality of shape components for upper and lower jaws with the Shapiro–Wilk’s test and a MANOVA was performed to detect differences between durophagous, non-durophagous, and fossil species, using an *a posteriori* pairwise comparison test. All tests were performed using *geomorph* package (Adams & Otarola-Castillo, 2013; Adams, Collyer & Sherratt, 2015) in R environment software (R Development Core Team, 2013).

**Table 2:** Summary of the results of the geometric morphometric analyses. Results from MANOVA for the PCs of the palate and lower jaws and distance probabilities from the pairwise comparison tests for the palate and lower jaws using three predefined groups (durophagous, non-durophagous and fossil turtles).

MANOVA	gl	SS/Wilks	*F*	*P*
Palate	2	0.43087	10.251	0.01*
LowerJaws	2	0.67515	11.248	0.01*

	Durophagous	Non-durophagous	Fossils
**Palate**			
Durophagous	1.00	0.01	0.32
Non-durophagous	0.01	1.00	0.01
Fossils	0.32	0.01	1.00
**Lower Jaws**			
Durophagous	1.00	0.01	0.30
Non-durophagous	0.01	1.00	0.01
Fossils	0.30	0.01	1.00

## Systematic Paleontology

**Table:** 

***Pleurodira*** Cope, 1864
***Pelomedusoides*** Cope, 1868
***Podocnemididae*** Cope, 1868

### Comment

Both Podocnemididae (Cope, 1868) and Podocnemidae (Baur, 1893) entered the literature in the 19th century and have been used since to designate the same biological group. The rules of the ICZN (1999) state that “A family-group name is formed by adding to the stem of the name [...] a suffix as specified in Article 29.2” (Article 29.1), that the suffix -IDAE is used for family names (Article 29.2), and that “If a generic name is or ends in a Greek or Latin word, or ends in a Greek or Latin suffix, the stem for the purposes of the Code is found by deleting the case ending of the appropriate genitive singular” (ICZN, 1999). For example, in the case of the genus *Archaeopteryx*, the genitive is Archaeopterygis and, thus, the stem for the purposes of the ICZN is Archaeopteryg-, giving the family name Archaeopterygidae; similarly, in the case of the genus *Homo*, the genitive is Hominis and the stem for the ICZN is Homin-, giving the family name Hominidae. In the case of the genus *Podocnemis*, formed by the greek words *podos*, foot, and *cnemis*, ocrea (Wagler, 1830), the Roman soldiers leg armour, the genitive case is Podocnemidos (D Amorim, F Lotti, E-E Kischlat, G Oliveira, pers. comm., 2015) and the stem for the purposes of the ICZN is Podocnemid-, giving the family name Podocnemididae. Additionally, Podocnemididae (Cope, 1868) has priority (Article 23.1) over Podocnemidae (Baur, 1893), and its usage (Article 29.5) is more common. As revealed by a Google Scholar search, “Podocnemididae” returns 1,020 and “Podocnemidae” 212 results. Accordingly, we chose to use the name Podocnemididae and advise future authors to do the same.

### *Stereogenyina* (Gaffney et al., 2011)

**Phylogenetic definition:***Stereogenyina* refers to the node-based clade that includes the most recent common ancestor of *Stereogenys cromeri* (Andrews, 1901), *Bairdemys hartsteini* (Gaffney & Wood, 2002), and *Cordichelys antiqua* (Andrews, 1903), and all its descendents.

**Diagnosis** (following Gaffney et al., 2011): Podocnemidids with a unique secondary palate among turtles, which is formed by maxillae and palatines and separated on the midline by a narrow cleft; no median maxillary ridge seen in other *Podocnemididae*; palate with a variable development of the rostral convexity and caudal concavity; palatine making up half or more of secondary palate; fossa orbitalis with extensive caudal pocket behind orbital rim enclosed by the septum orbitotemporale.

### *Bairdemys* (Gaffney & Wood, 2002)

**Derivatio nominis:** For Donald Baird (see Gaffney & Wood, 2002).

**Type species:***Bairdemys hartsteini* (Gaffney & Wood, 2002).

**Diagnosis:** Stereogenyins with an extremely small and slitlike antrum postoticum entrance; a ventral vertical flange on the squamosal; a distinct caudal projection of the squamosal; a deep convexity on the triturating surface; medial edges of palatal cleft curved; eustachian tube separated by bone from fenestra postotica; both foramina nervi-hypoglossi combined and recessed in a short canal opening on occipital surface; jugal-pterygoid contact; pectoral-abdominal sulcus not crossing mesoplastron; pectoral-humeral sulcus in cranial half of entoplastron; and intergular scales barely extending onto entoplastron.

***Bairdemys thalassica*** sp. nov.

**Derivatio nominis:** From the Greek word *thalassa* (= sea).

**Holotype:** IVIC-P-2908 (IVIC-P: Colección de Paleontología, Instituto Venezolano de Investigaciones Científicas, Caracas, Venezuela), almost complete skull, lacking the pre-orbital region, the right portion of the skull roof, and the right lateral wall of the fossa temporalis.

**Locality:** El Miedo Cave (10°51′26″ N; 68°36′41″ W), located 15.5 km southwest of Yaracal town, Cerro Misión (Fig. 1), Falcón State, Venezuela.

**Geology:** The Capadare Formation is a thick sequence of massive carbonate layers deposited in shallows marine waters (Lorente, 1978), which crops out in northwestern Venezuela. It represents isolated carbonate platforms, without coastal or continental influence, in entirely open sea conditions, with well oxygenated and clear waters, normal salinity, and moderate energy in a tropical climate (Lorente, 1978; Díaz de Gamero, 1985). Development of karstic systems is common in the Capadare Formation, as exemplified by the well-known El Miedo and Zumbador caves. Based on its foraminifer assemblage, the Capadare Formation was assigned to the middle Miocene, *Globorotalia fohsi fohsi*-*Globorotalia sjakensis* Biozone (Renz, 1948; Lorente, 1978; Díaz de Gamero, 1985). Using strontium isotope analyses (ratio of ^87^Sr/^86^Sr) from a marine mollusc shell (*Ostrea* sp.), (Solórzano & Rincón, in press) placed the Capadare Formation in the middle Miocene (∼12.24 Ma). The record of vertebrates from the Capadare Formation includes *Pelagornis* cf. *P. chilensis* (Aves: Odontopterygiformes) and shark teeth (Rincón & Stucchi, 2003; Solórzano & Rincón, in press), but Pleistocene fossils were also recovered in the bottom of the El Miedo cave (Rincón, 2003; Rincón & White, 2007; Montellano-Ballesteros, Rincón & Solórzano, 2014).

**Diagnosis:** A *Bairdemys* with a short midline contact of the pterygoids; a longer basisphenoid-quadrate contact; a more rounded rostral tip of the basisphenoid; dermal scale iv expanding onto the postorbital, quadratojugal, and parietal; and a slitlike opening of the canal containing both foramina nervi hypoglossi significantly smaller than the foramen jugulare posterius.

### Description

**Figure 2:**
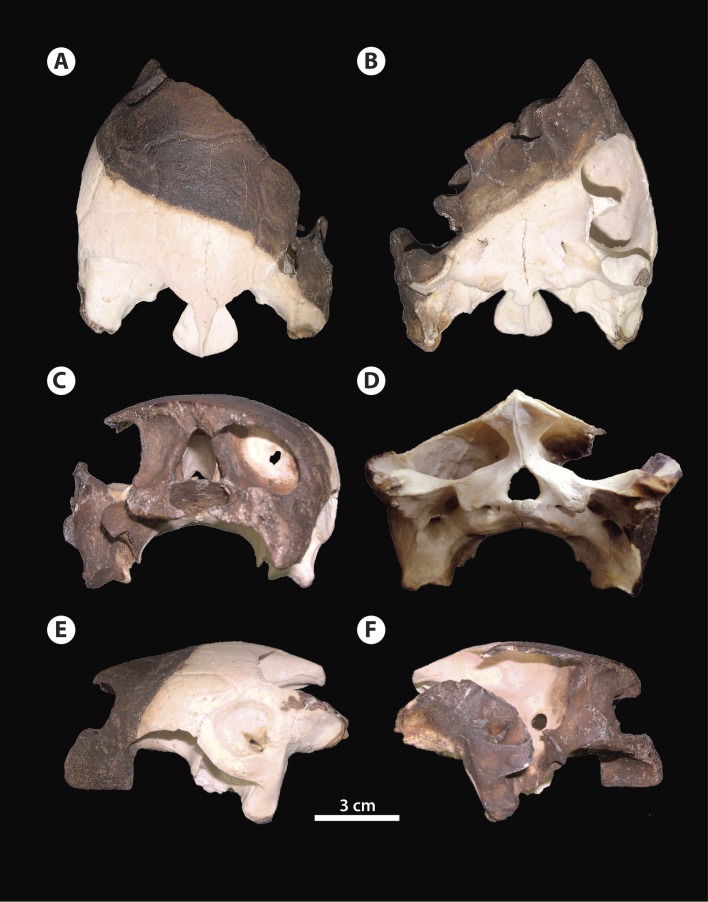
*Bairdemys thalassica* sp. nov. Holotype skull IVIC-P-2908 in (A) dorsal, (B) ventral, (C) rostral, (D) caudal, (E) left, and (F) right lateral views.

**Figure 3:**
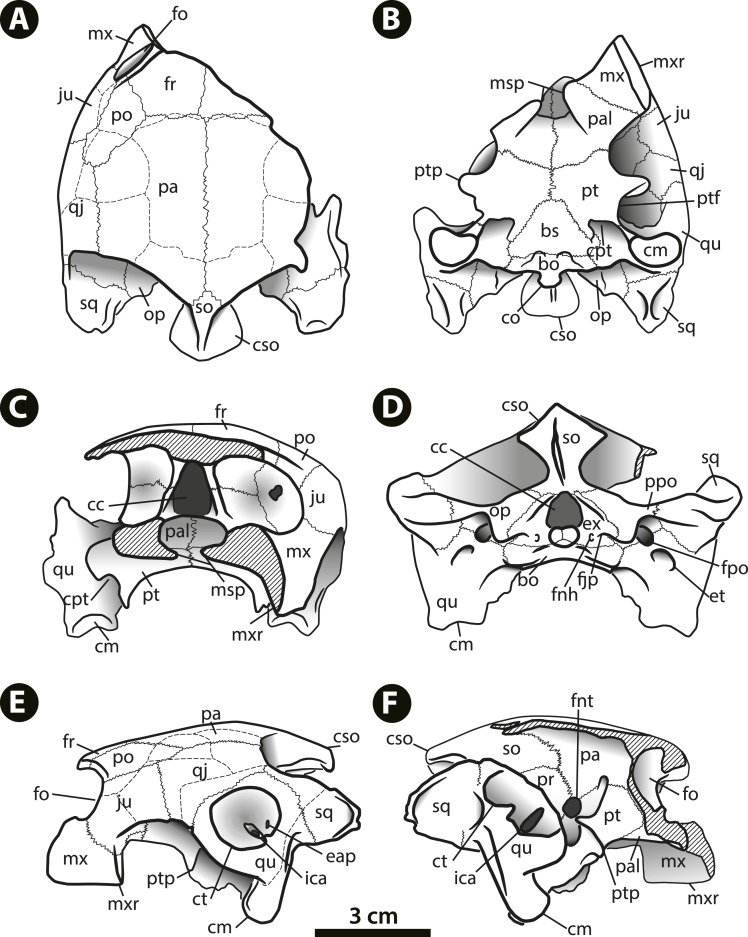
*Bairdemys thalassica* sp. nov. Drawing of the holotype skull IVIC-P-2908 in (A) dorsal, (B) ventral, (C) rostral, (D) caudal, (E) left, and (F) right lateral views. **Abbreviations: bo,** basioccipital; **bs,** basisphenoid; **cc,** cavum cranii; **cm,** condylus mandibularis; **co,** condylus occipitalis; **cpt,** cavum pterygoidei; **cso,** crista supraoccipitalis; **ct,** cavum tympani; **eap,** entrance of the antrum postoticum; **et,** eustachian tube; **ex,** exoccipital; **fjp,** foramen jugulare posterius; **fnh,** foramina nervi hypoglossi; **fnt,** foramen nervi trigemini; **fo,** fossa orbitalis; **fpo,** fenestra postotica*;* **fr,** frontal; **ica,** incisura columellae auris; **ju,** jugal; **msp,** midline cleft of the secondary palate; **mx,** maxilla; **mxr,** maxillary ridge; **op,** opisthotic; **pa,** parietal; **pal,** palatine; **po,** postorbital; **ppo,** processus paroccipitalis; **pt,** pterygoid; **ptf,** pterygoid flange; **ptp**, processus trochlearis pterygoidei; **qj,** quadratojugal; **qu,** quadrate; **so,** supraoccipital; **sq,** squamosal.

**Skull**—The holotype and only specimen assigned to *Bairdemys thalassica*, IVIC-P-2908 (Figs. 2 and 3), is very well preserved, with almost no taphonomic distortion. It is nearly complete, lacking only the rostral and the right dorsal regions. Only the vomeri, the premaxillae, and the prefrontals are completely absent. The frontal, jugal, quadratojugal, parietal, squamosal, maxilla, and palatine have one of the elements of the pair entirely or partially missing, but the other is complete, allowing a detailed description.

Among *Bairdemys* species, the general shape of the skull of *B. thalassica* is more similar to that of *B. venezuelensis*, which is rounded, almost oval in dorsal outline (Fig. 2A), and relatively high in lateral view (Figs. 2C and 2D). The temporal emargination is not well developed, and rostrocaudally shallower than in *B. hartsteini* and *B. sanchezi* (Gaffney et al., 2008). The cheek emargination is as developed as in other *Bairdemys*, not reaching the dorsal edge of the cavum tympanii (Fig. 2E). As in other *Bairdemys* species, the orbits face laterally and the skull bone surface is smooth, lacking ornamentations except for the dermal scale sulcii.

**Dermal scales of the skull**—The scale sulcii of the skull are well defined in IVIC-P-2908. In comparison to those of Meiolaniformes, the only turtle lineage with a described and systematized scale pattern (Simpson, 1938; Gaffney, 1996; Sterli & de la Fuente, 2011; Sterli & de la Fuente, 2013; Rabi et al., 2014 also adopted this system to describe the scales of the eucryptodiran *Annemys levensis*), *B. thalassica*, as well as all pelomedusoids with preserved sulcii, shows a great reduction in the number of scale units. For example, *Mongolochelys efremovi* (Khosatzky, 1997), bears 14 types of scales on the skull (Sterli & de la Fuente, 2013), whereas pelomedusoids have only between 6 and 8 types. This hampers tracing scale homology between those groups, and a more in depth analysis, also including cryptodires and stem-*Testudines*, is needed to fully establish homology relations of the turtle skull dermal scales. For now, we decided to create a new system to identify the *Pelomedusoides* scales, using lower-case latin numbers. Potencial homologues with the proposed Meiolaniformes system (herein called SF13) are mentioned in the description.

**Figure 4:**
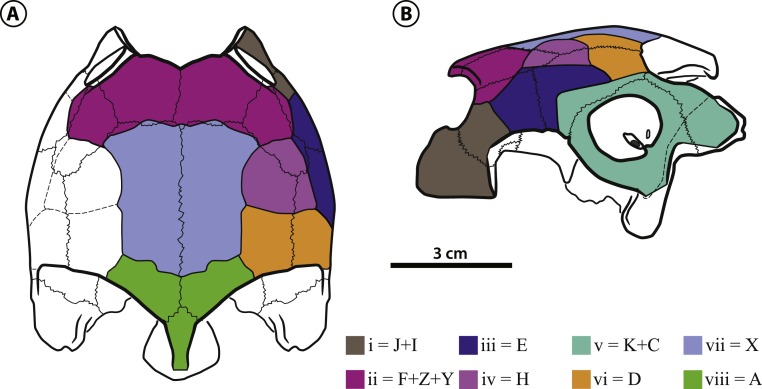
*Bairdemys thalassica* sp. nov. Dermal scales of the skull in (A) dorsal and (B) lateral view. On the identification of each scale (in lower case latin numbers, from i to viii) there is after the “=” mark the preliminary homology assessment in relation to the Sterli & de la Fuente (2013) system (see *Dermal scales of the skull* section).

The scales i, iii, v, vi and iv (Fig. 4) are paired in *Bairdemys thalassica* and in all studied *Pelomedusoides*. Scales iii can be divided in two units in some pelomedusoids, e.g., *Pelomedusa subrufa* (Bonnaterre, 1789)*, Erymnochelys madagascariensis*, and *Podocnemis vogli* (Müller, 1935), or absent, e.g., *Podocnemis sextuberculata* (Cornalia, 1849), and *Peiropemys mezzalirai* (Gaffney et al., 2011). Scales ii, vii and viii are unpaired in *B. thalassica*, as well as in most pelomedusoids, e.g., *Peltocephalus dumerilianus*. In some taxa, e.g., *Pelomedusa subrufa*, *Podocnemis unifilis*, and *Neochelys fajumensis* (Andrews, 1903), scale ii is paired. Scale viii is absent in *Pelomedusa subrufa* and *Cordichelys antiqua*, the. Scale i forms the ventral-most portion of the rostral edge of the skull, covering the premaxilla, maxilla, and a portion of the jugal. It contacts scales ii, dorsally (caudal to the orbit), and iii, caudally. This scale can be the homologue of scales J plus I of SF13. A large and single ii scale covers the prefrontal, frontal, and portions of the postorbital and jugal, and contacts scales iii lateroventrally, iv laterocaudally, and vii caudomedially. We suggest that scale ii of *Pelomedusoides* can be a total or partial fusion of scales F, Z, and Y of SF13. Scale iii covers parts of the jugal, postorbital and quadratojugal in the lateral region of the skull. In contrast to the condition in *Peltocephalus dumerilianus*, *E. madagascariensis*, and *Podocnemis vogli*, scale iii in *B. thalassica* does not reach the orbital margin. This condition is shared with *Bairdemys hartsteini* and may represent a synapomorphy of *Bairdemys*. Scale iii contacts scales iv, medially, v, laterocaudally and vi, caudally, and we suggest that it corresponds to scale E in SF13. Over parts of the postorbital, quadratojugal and parietal of *B. thalassica* lies a semi-circular paired scale iv, that contacts scales vii medially, iii laterally and vi caudally. This scale is not found in any of the other analyzed pelomedusoids and may be the homologue to scale H of SF13. Scale v is relatively large and covers the quadrate, squamosal and parts of the quadratojugal, contacting scale vi medially. In this region, there are two scales in meiolaniforms, K and C (Sterli & de la Fuente, 2013), and we suggest that both are fused into scale v of *Pelomedusoides*. Due to the presence of scale iv, scale vi is reduced in *B. thalassica* compared to the condition of other pelomedusoids, e.g., *Peltocephalus dumerilianus*. It contacts scales vii, medially, and viii, caudomedially. Scale vii (identified otherwise as “interparietal scale”) has parallel lateral margins and covers half of the parietals and a small portion of the frontals, contacting scale viii caudally. Scale viii is also unpaired and covers a small caudal portion of the parietals and supraoccipital. We suggest that scales vi, vii, and viii are respectively homologues of scales D, X, and A of SF13.

**Figure 5:**
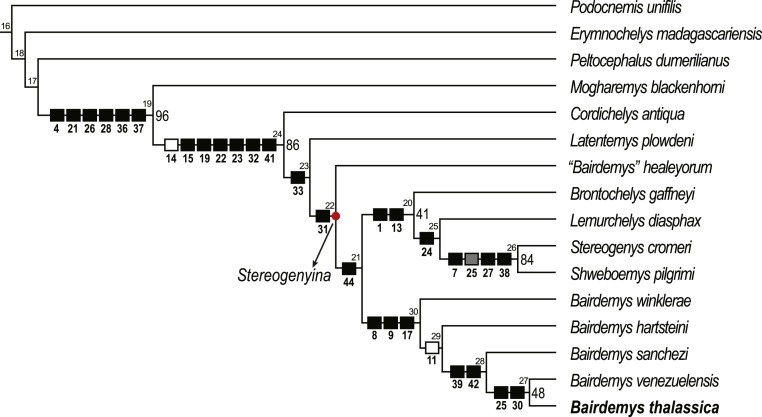
Phylogenetic relations of *Stereogenyina* based on the single most parsimonious tree obtained in the present study. Node numbers are indicated above each node and GC bootstrap values above 40 in front of them. Squares indicate the synapomorphies of the respective clade (black = 0 to 1, gray = 0 to 2, white = 1 to 0).

**Figure 6:**
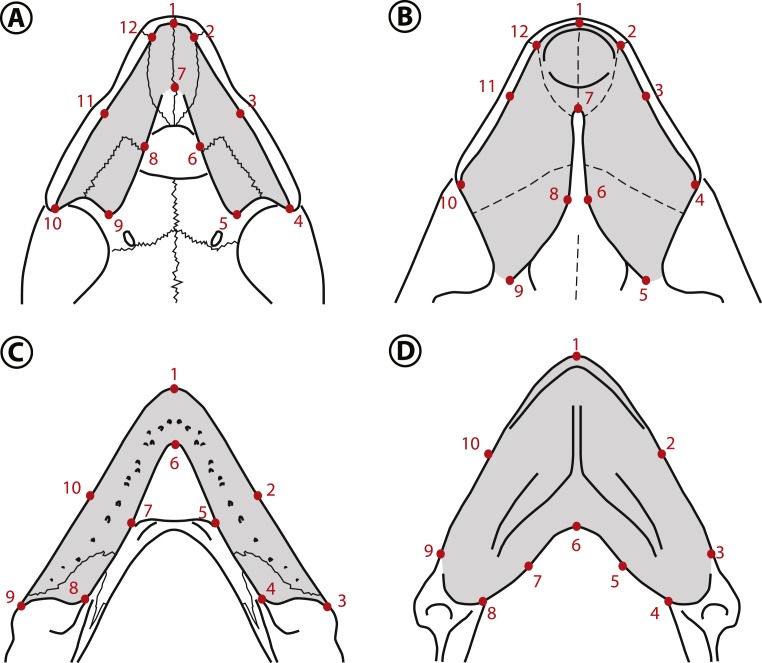
Landmarks used in the geometric morphometric analyses. Landmarks shown in the palates of (A) *Peltocephalus dumerilianus* and (B) *Bairdemys winklerae*, and in the lower jaws of (C) *Peltocephalus dumerilianus* and (D) *Bairdemys venezuelensis*. For the description of the landmarks see Supplemental Information 3.

**Figure 7:**
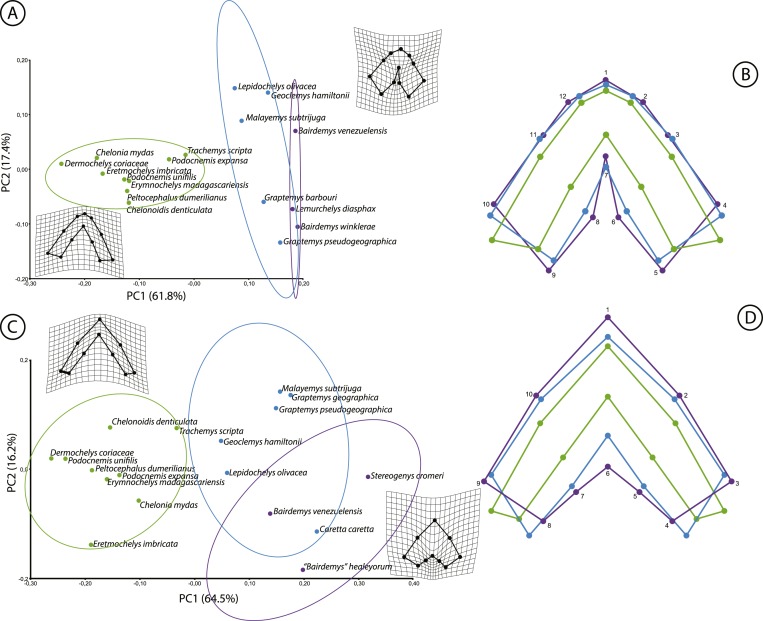
Results of the geometric morphometric analyses. Principal component analysis (A and C) derived from the first two principal components (PC1 and PC2) and comparison of the mean values of the coordinates (B and D) for the upper (A and B) and the lower jaws (C and D). D’Arcy Thompson grids correspond to extreme shape variation. Durophagous taxa are represented in blue, non-durophagous in green, and fossil taxa with unknown diet in purple.

**Frontal**—Each frontal forms the caudomedial portion of the orbital ridge, and is sutured with the postorbital laterocaudally, parietal caudally, and with its counterpart medially. The curved skull outline in lateral view in the prefrontal-frontal contact region, characteristic of *Bairdemys* (Gaffney & Wood, 2002) cannot be seen in IVIC-P-2908, because it is usually located rostral to the preserved portion of the frontal. As in all *Bairdemys* species the frontal is very thick and also has a thick parasagittal process that contacts the processus inferior parietalis and the ascending process of the palatine (Fig. 3C); this separates the sulcus olfactorius from the fossa orbitalis.

**Parietal**—As in all *Podocnemididae*, each parietal is composed of a horizontal plate and a descending process, the processus inferior parietalis (Gaffney & Wood, 2002). In dorsal view, the horizontal plate contacts the frontal rostrally, postorbital laterorostrally, quadratojugal laterally, supraoccipital caudomedially, and the other parietal medially. The processus inferior parietalis is a very thick structure, as in *Stereogenys cromeri*, *Shweboemys pilgrimi,* and other *Bairdemys* species (Gaffney & Wood, 2002). It forms the roof and lateral wall of the cavum cranii and, together with the postorbital, the caudal surface of the septum orbitotemporale. The processus contacts the medial and lateral ascending processes of the pterygoid caudomedially, forming the roof of the sulcus palatinopterygoideus (Figs. 2F and 3F). Caudal to this sulcus, the parietal forms the dorsal margin of the foramen nervi trigemini.

**Jugal**—As in all pelomedusoid turtles, the jugal forms the laterocaudal portion of the orbital ridge, between the maxilla and the postorbital (Gaffney & Wood, 2002). The semi-circular dorsal margin of the cheek emargination is formed by the jugal rostrally and the quadratojugal caudally, and the contact between this two bones is an almost vertical line. The jugal-maxilla contact is nearly horizontal as in *B. sanchezi* and *B. venezuelensis*. As in most *Stereogenyina*, the jugal of *B. thalassica* does not enter the palatal triturating surface. In the septum orbitotemporale, it contacts the postorbital dorsomedially, the pterygoid medially, and the palatine ventrally, differing only from *Stereogenys cromeri* and *Shweboemys pilgrim*, among *Stereogenyina* in which the palatine prevents the jugal-pterygoid contact.

**Quadratojugal**—The quadratojugal contacts the jugal rostrally, the postorbital rostromedially, the parietal medially, the quadrate laterally, and the squamosal, in a very thin process (Fig. 3E). This arrangement is shared with all pelomedusoids, except for *Podocnemis* spp. and *Cerrejonemys wayuunaiki* (Cadena, Bloch & Jaramillo, 2010), in which there is no quadratojugal-postorbital contact, due to the great reduction of the postorbital (Gaffney & Wood, 2002; Cadena, Bloch & Jaramillo, 2010). As in all pelomedusoids the quadratojugal-quadrate suture is curved, following the margin of the cavum tympani (Fig. 3E).

**Squamosal**—The squamosal contacts the quadratojugal rostrodorsally, on the lateral edge of the temporal emargination, the opisthotic medially, and the quadrate rostrally, in a long suture. A dorsal process forms the laterocaudal portion of the wall of the fossa temporalis superior. As in all *Testudines* the squamosal of *B. thalassica* encloses a cavity caudodorsal to the cavum tympani, the antrum postoticum (Gaffney, 1979). In *Bairdemys* spp., *Lemurchelys diasphax* (Gaffney et al., 2011), and *Stereogenys cromeri* the opening of the antrum postoticum in the cavum tympani is extremely reduced and slit-shaped. The squamosal in *Bairdemys* spp. projects more caudally than in any other *Stereogenyina*. In *B. thalassica* its outline in dorsal view is more rounded caudally, aproaching the condition of *B. hartsteini* and differing from the squamosal of the other species of *Bairdemys*, e.g., *B. winklerae* (Gaffney et al., 2008), which have a sharp ending. The dorsomedial and ventrolateral surfaces of the caudal projection of the squamosal were probably attachment sites for the Musculus (M.) adductor mandibulae and the M. depressor mandibulae, respectively. Both of them are more developed in *B. thalassica* than in any other known *Stereogenyina*.

**Postorbital**—The postorbital is composed of a dorsal horizontal plate, very thick in *Bairdemys* spp., and a ventral vertical process that forms most of the septum orbitotemporale (Figs. 2C and 3C). The horizontal plate forms the caudal portion of the orbital ridge and contacts the frontal medially, parietal caudomedially, quadratojugal laterocaudally, and jugal laterally. The ventral process contacts the palatine rostrally and the pterygoid and jugal caudally, as seen in all other *Bairdemys* species, as well as in *Lemurchelys diasphax* and *Latentemys plowdeni* (Gaffney et al., 2011).

**Quadrate**—The quadrate forms the entire cavum tympani which encloses the incisura columellae auris and the opening of the antrum postoticum (Figs. 2E and 3E). As in other *Bairdemys* species (Gaffney & Wood, 2002; Gaffney et al., 2008) the fossa precolumellaris is absent in *B. thalassica*. The incisura columellae auris is completely surrounded by bone and encloses the *stapes* and the eustachian tube. In *B. thalassica* this bone forms a very thick caudal bar, as seen in all *Stereogenyina* that have this region preserved (Gaffney & Wood, 2002). The fenestra postotica is separated from the eustachian tube (Fig. 3D), as in all *Bairdemys* species and *Latentemys plowdeni* (Gaffney et al., 2011). The rostral and dorsal surfaces of the quadrate of *B. venezuelensis* and *B. hartsteini*, which form the caudal wall of the fossa temporalis inferior and the floor of the fossa temporalis superior, respectively, bears a small crest previously known only in those two species, in the same region where the processus trochlearis oticum of cryptodires is found (Gaffney & Wood, 2002). In *B. thalassica*, this structure is also present and, perhaps due to its excellent preservation, an additional crest is seen parallel to the former one, forming a smooth groove between them. The function of this structure is unknown.

In the wall and floor of the fossa temporalis, the quadrate contacts the pterygoid rostroventrally, the prootic rostrodorsally, the opisthotic caudomedially, and the squamosal caudally and laterocaudally. Together with the prootic, it forms the margins of the foramen stapedio-temporale, which opens rostrodorsally as in other podocnemidids. On its ventral surface, the quadrate contacts the basioccipital caudomedially, the basisphenoid medially, and the quadrate ramus of the pterygoid rostrally, close to the condylus mandibularis (Figs. 2B and 3B). The condylus has a concave rostral margin, as in other *Bairdemys* species, contrasting with the condition of *Stereogenys cromeri* and *Lemurchelys diasphax*, in which this margin is straight (Gaffney et al., 2011). The condylus mandibularis of *B. thalassica* is in the same line as the basisphenoid-basioccipital contact, a position closer to that seen in *Cordichelys antiqua*, *Stereogenys cromeri*, and *B. venezuelensis* than to that of other *Bairdemys* species. The quadrate forms the caudal portion of the roof of the cavum pterygoidei (Fig. 3B), which is also formed by the basisphenoid and pterygoid. On its lateral surface, around the cavum tympani, the quadrate contacts the quadratojugal rostrally and the squamosal caudally. On the caudal surface, it contacts the squamosal laterodorsally, the opisthotic dorsomedially, the exoccipital medially, and the basioccipital ventromedially. Finally, it forms the lateral and vetral margins of the fenestra postotica.

**Maxilla**—The maxila contacts the palatine caudomedially and the jugal dorsocaudally (Figs. 3B and 3E). The dorsal process that contacts the frontal in *B. venezuelensis* and *B. hartsteini* (Gaffney & Wood, 2002) is replaced by a process of the palatine in *B. thalassica* (Fig. 3C). The maxilla contributes less to the orbital floor in comparisson to that of other *Bairdemys* species, forming only its rostrolateral portion, where it contacts the jugal caudally and the palatine medially, as in *Brontochelys gaffneyi* (Wood, 1970) and *Podocnemis expansa* (Schweigger, 1812). On the palatal triturating surface, the suture with the palatine extends laterally, ending caudal to the end of the labial ridge (Fig. 3E). This condition is different from that of *B. hartsteini*, in which this suture ends more medially. In comparison to other *Stereogenyina*, the labial ridge in *Bairdemys* sp. is high and thin, as in *Cordichelys antiqua*.

**Palatine**—As in other *Stereogenyina* (Gaffney & Wood, 2002) the palatine of *B. thalassica* is a very complex bone. It bears two distinct horizontal plates forming the caudal portions of the primary and secondary palate, and two very thick vertical plates, one separating the fossa orbitalis from the sulcus palatinopterygoideus and another forming the rostromedial wall of the sulcus palatinopterygoideus and the laterorostral wall of the cavum cranii (Figs. 2F and 3F). The horizontal plates are sutured to the maxilla rostrally and to the pterygoid caudally. These form the caudal portion of the midline cleft and the triturating surface, which extends towards the palatine-pterygoid contact, but does not reach it as in *Stereogenys cromeri*, *Shweboemys pilgrimi*, *Brontochelys gaffneyi*, and *Lemurchelys diasphax*. Close to this contact there is a tiny aperture identified as the foramen palatinum posterius. This is usually much reduced and sometimes absent in *Stereogenyina*. The palatine forms most of the fossa orbitalis floor, where it contacts the maxilla laterorostrally, the jugal laterocaudally, and the postorbital caudally. The horizontal plate also forms the rostral most part of the floor of the sulcus palatinopterygoideus.

The typical *Stereogenyina* secondary palate, (unique among *Pleurodira*) is well developed in *B. thalassica*, but less extensive than in *Lemurchelys*, *Stereogenys*, and *Shweboemys* (Gaffney et al., 2011). Although it is not completely preserved, the edges of its midline cleft are curved, as in other *Bairdemys* species, *Cordichelys antiqua*, and *Latentemys plowdeni*; in contrast to the straight/parallel edges found in *Stereogenys*, *Shweboemys*, *Lemurchelys*, and *Brontochelys*.

The lateral vertical plate is a dorsal process of the palatine, medial to the jugal and rostral to the ascendent process of the pterygoid. It contacts the ventral parasagittal process of the frontal in all species of *Bairdemys*, separating the fossa orbitalis from the sulcus palatinopterygoideus. As in all *Stereogenyina,* this process is well developed and becomes thicker rostrally. The medial vertical plate contacts the pterygoid caudally and the parietal dorsally, forming the wall that separates the cavum cranii from the sulcus palatinopterygoideus.

**Pterygoid**—As the palatine and quadrate, the pleurodiran pterygoid is a very complex bone. It is composed of a ventral horizontal plate that in *Bairdemys* species contacts the palatine rostrally, the quadrate laterocaudally, and the basisphenoid caudally. As is typical of *Pleurodira* (Gaffney, 1979), the pterygoid has a laterally expanding processus trochlearis pterygoidei, forming a right angle to the horizontal plate (Figs. 3B and 3F). Caudal to the base of the process, the pterygoid flange is well developed, covering the cavum pterygoideum as in all podocnemidids. This flange is a very thin sheet of bone and is frequently broken in fossils. In IVIC-P-2908 only parts of the left flange are preserved, but it is possible to infer that its shape and extension are similar to those of other species of *Bairdemys*.

Two dorsal processes are seen rostral and medial to the processus trochlearis pterygoidei. The rostral process contacts the jugal rostrally, the postorbital rostrodorsally and the parietal dorsally. The medial process is parallel to the processus trochlearis pterygoidei, forming the caudoventral portion of the sulcus palatinopterygoideus. It contacts the parietal rostrodorsally and the prootic caudodorsally, which together form the margins of the foramen nervi-trigemini opening laterally to the cavum cranii (Fig. 3F).

As typical of *Podocnemoidae*, the cavum pterygoidei of *B. thalassica* lies on the caudal portion of the ventral surface of the pterygoid. As in other *Bairdemys* species, *Cordichelys antiqua*, and *Stereogenys cromeri*, this structure is formed mainly by the pterygoid and quadrate with a small laterocaudal contribution of the basisphenoid. The extension of the medial contact between the pterygoids in *B. thalassica* is similar to that of *B. venezuelensis* but shorter than in *B. sanchezi*. The sutures of this region are not preserved in *B. hartsteini* and *B. winklerae*.

**Supraoccipital**—The supraoccipital forms the dorsal margin of the foramen magnum and its contacts are very similar in all *Pelomedusoides*: rostrodorsally to the parietals, lateroventrally to the prootic, caudally and lateroventrally to the opisthotic, and caudoventrally to the exoccipital, the latter on a narrow suture. In *B. thalassica* it has well developed horizontal plates that extend laterally to the crista supraoccipitalis, a common feature among *Stereogenyina* (Gaffney et al., 2011). In *Bairdemys* spp. those plates are even more pronounced and form a bulbous structure on the caudal edge of the crista supraoccipitalis (Figs. 2A and 3A). The crista supraoccipitalis of *B. thalassica* is not as expanded as in *B. venezuelensis* and *B. winklerae*, and does not reach the line formed by the caudal edges of the squamosals.

**Exoccipital**—The exoccipitals form the lateral and ventral margins of the foramen magnum and meet the supraoccipital dorsally. They also form the laterodorsal portion of the condylus occipitalis, where they contact the basioccipital ventrally (Fig. 3D). Each exoccipital contacts the opisthotic laterodorsally, the basioccipital along its entire ventral margin and its counterpart on the bottom of the foramen magnum. The foramen jugulare posterius (Fig. 3D) is formed mainly by the exoccipitals with small contributions of the basioccipital ventrally and opisthotics laterally. Dorsal to this foramen, each exoccipital and opisthotic form a covering. This develops into a trough extending laterally from this foramen (Gaffney et al., 2008), as seen in *B. venezuelensis* and *B. sanchezi*. As in all Stereogenyini (*sensu* Gaffney et al., 2011) both foramina nervi-hypoglossi are combined and recessed into a single short canal, which opens laterocaudally, and is located between the condylus occipitalis and the foramen jugulare posterius. In *B. thalassica* the single opening of the canal containing both foramina nervi-hypoglossi is slit-shaped and substantially smaller than the foramen jugulare posterius , an autapomorphy of the new species.

**Basioccipital**—The basioccipital of *B. thalassica* contacts the basisphenoid rostroventrally, the quadrate lateroventrally, the opisthotic laterodorsally, and the exoccipital caudodorsally. It forms the ventral portion of the condylus occipitalis, parts of the foramina jugulare posterius and anterius, as well as part of the cavum labyrinthicum floor. The contact between the basioccipital and basisphenoid is convex rostrally, as in other species of *Bairdemys* (Gaffney & Wood, 2002) and *Stereogenyina*, except in *Latentemys plowdeni* (Gaffney et al., 2011).

**Prootic**—The prootic contacts the parietal rostrodorsally, the supraoccipital caudodorsally, the opisthotic caudally, the quadrate laterally, and the pterygoid rostroventrally. It forms the medial edge of the foramen stapedio-temporale, which in *B. thalassica* and other *Stereogenyina* (Gaffney & Wood, 2002) opens more rostrally than in *Podocnemis* spp. At its rostral margin, between the pterygoid and parietal contact, it forms the caudal edge of the foramen nervi trigemini (Fig. 3F).

**Opisthotic**—The opisthotic contacts the prootic rostrodorsally, the supraoccipital laterodorsally, the quadrate laterorostrally, and the exoccipital caudomedially. At its laterocaudal margin, the processus paraoccipitalis meets the squamosal dorsally and, ventral to this process, the opisthotic contacts the basioccipital. It encloses the hemispherical cavity of the recessus labyrinthicus opisthoticus (Gaffney, 1979) and forms the caudal and dorsal walls of the cavum labyrinthicum and the rostral edge of the foramen jugulare anterius. As in other species of *Bairdemys* (Gaffney & Wood, 2002; Gaffney et al., 2008), the opisthotic of *B. thalassica* also forms the roof of the cavum acustico-jugulare, the dorsomedial margin of the fenestra postotica, and the laterodorsal margin of the foramen jugulare posterius. Lateral to the later foramen, a trough extends laterally towards the fenestra postotica. In *B. thalassica*, as well as in *B. venezuelensis* and *B. sanchezi*, this trough is deep, in contrast to the shallower trough of other *Stereogenyina* (Gaffney et al., 2008).

**Basisphenoid**—The basisphenoid of *B. thalassica* is subtriangular and has a rounded rostral edge (Figs. 2B and 3B), as seen in *B. venezuelensis*, and in contrast to the angular edge of *B. hartsteini* and *B. sanchezi* (Gaffney et al., 2008). The lateral margins of the bone are straight, differing from the curved lateral margins as seen in *B. sanchezi*. It contacts the pterygoid rostrally, the quadrate laterally, and the basioccipital caudally. The basisphenoid forms a small part of the medial surface of the cavum pterygoidei, where the foramen anterius canalis carotici interni opens laterally.

## Results

### Phylogenetic analysis

A single MPT, 83 steps long, with CI = 0.723 and RI = 0.826, was found (Fig. 5) and it supports the assignment of *B. thalassica* to the genus *Bairdemys* (Fig. 5). A *Bairdemys* clade was recovered (node 30, Fig. 5) exclusive of *“Bairdemys” healeyorum* (Weems & Knight, 2013). The latter taxon was positioned as sister to the Stereogenyita + *Bairdemys* clade (node 21, Fig. 5), as supported by a smooth rostral tip of the lower jaw, lacking a rostral hook (car. 44, 0 → 1). In a recent phylogenetic analysis (Menegazzo, Bertini & Manzini, 2015), the only one to include *“B.” healeyorum*, that taxon was placed as sister to *B. venezuelensis* + *B. hartsteini*. In order to test this possibility, we enforced the position of *“B.” healeyorum* inside *Bairdemys*, resulting in 30 MPTs 84 steps long, the strict consensus of which includes a politomy with all the *Bairdemys* species. The comparison of the two topologies using the Templeton test (Templeton, 1983) yields a significant *p* value of 0.0021 (Table 1), supporting the position of *Bairdemys healeyorum* outside the *Bairdemys* clade. However, we chose not to propose a new genus for this taxon, waiting for further analyses to confirm our results. Because of its very incomplete condition, we did not include *Bairdemys miocenica* (Collins & Lynn, 1936), the new combination of *“Taphrosphys” miocenica* proposed by Weems & Knight (2013), in the analysis. Yet, the authors used comparisons to the plastron morphology of *Bairdemys venezuelensis* and *“B.” healeyorum* to include that taxon in the *Bairdemys* clade, and our analysis supports a more widespread distribution of those characteristics across the *Stereogenyina* lineage. Accordingly, we consider *“B.” miocenica* a *Stereogenyina* incertae sedis.

**Figure 8:**
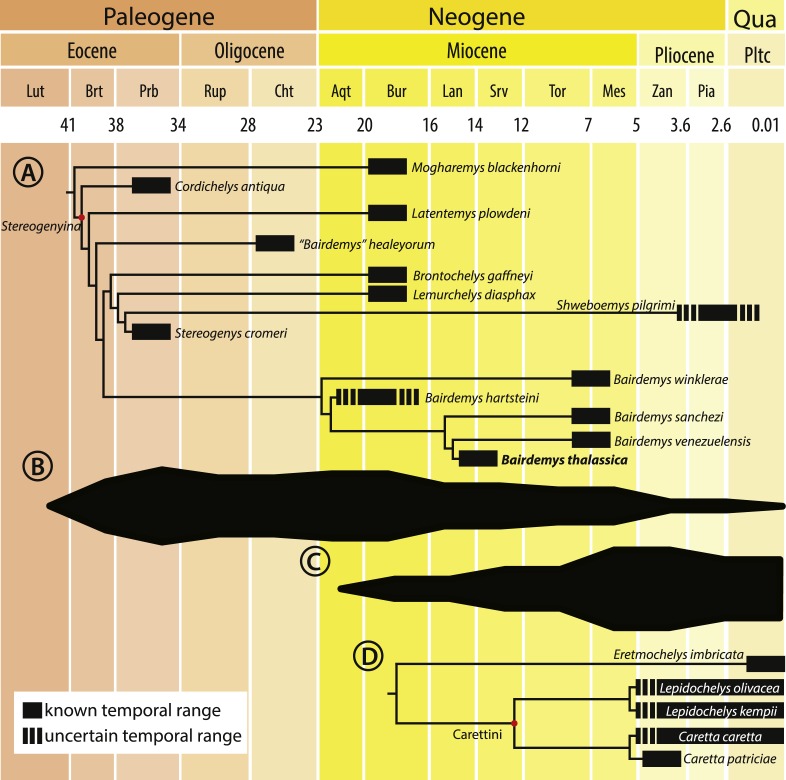
Cladogram calibrated on a time scale and species diversity plot. Cladogram calibrated on a time scale (A and D) and species diversity plot based on the count of lineages on each age (B and C), from the Eocene to the present, for *Stereogenyina* (A and B) and Carettini turtles (C and D, cladogram based on Parham & Pyenson, 2010). Layout modified from Romano et al. (2014)).

*Bairdemys thalassica* is closer to *Bairdemys venezuelensis* (node 27, Fig. 5) than to the other species of *Bairdemys*, sharing a moderately developed (caudally) palatine that reduces the contact between the pterygoids (car. 25, state 0 → 1) and a condylus mandibularis caudal or at the same line of the basioccipital-basisphenoid contact (car. 30, state 0 → 1). *B. hartsteini* is the sister taxon to this clade, followed by *B. sanchezi* and *B. winklerae*. This arrangement implies an early Miocene (Fig. 8A), rather than Oligocene (Weems & Knight, 2013) origin of *Bairdemys*.

The Stereogenyita clade (node 20, Fig. 5) was recovered with the same arrangement as in Gaffney et al. (2011), with *Brontochelys gaffneyi* and *Lemurchelys diasphax* as successive sister-taxa to the *Stereogenys cromeri* plus *Shweboemys pilgrimi* clade. Stereogenyita is supported by a very wide pre-orbital region of the skull (car. 1, state 0 → 1) and a low labial ridge (car. 13, state 0 → 1). In contrast to Gaffney et al. (2011), *Cordichelys antiqua* and *Latentemys plowdeni* were not recovered as closer to *Bairdemys* than to Stereogenyita, but as successive sister taxa to the *“B.” healeyorum* + (Stereogenyita + *Bairdemys*) clade (node 22, Fig. 5). The latter clade is supported by a condylus mandibularis slightly wider than longer, with a “kidney bean” shape and a convex caudal margin (car. 31, state 0 → 1), and implies that the secondary palate with parallel edged midline cleft, synapomorphic for Stereogenyita clade, is derived from a secondary palate with curved-edged midline cleft, as in *Cordichelys antiqua* and *Latentemys plowdeni*.

### Morphometric analysis

The estimated shape measurement error was 2.4% for the upper and 5.2% for the lower jaws total variance. The coordinate data from the upper and lower jaws are normally distributed (Shapiro–Wilk test resulted in *w* = 0.9778 and 0.9772, respectively). The shape variation was decomposed in ten and eight Principal Components (PCs) for the upper and lower jaws, respectively. For the upper jaw, the first two PCs accounted for 79.2% of the variation, and for the lower jaw the first two PCs explained 80.7% of the total variation in shape.

The main variation (PC1 = 61.8%) in the shape of the upper jaw in the analyzed species is related to the expansion to the midline or the lateral reduction (Fig. 7A) of the caudomedial edges of the triturating surfaces (landmarks 5, 6, 8, and 9). The variation accounted for in the PC2 (17.4%) is related to the rostrocaudal displacement of the caudomedial edge (landmark 7). The shape of the lower jaws changed mainly (PC1 = 64.5%) in the rostrocaudal displacement of the caudomedial edge (landmark 6) and the caudomedial-rostrolateral displacement of the medial edge of the triturating surface (landmarks 5 and 7). The PC2 (16.2%) accounted for the variation in the caudal edge of the medial portion of the triturating surface (landmarks 4 and 8) in the rostromedial-caudolateral axis (Fig. 7C). Considering the *a priori* defined groups (non-durophagous, durophagous and unknown), the changes in the upper and lower jaws shape of the durophagous and the unknown (*Stereogenyina*) groups when compared to the non-durophagous is characterized by a medial and caudal expansion of the triturating surfaces.

## Discussion

### Feeding habits of Stereogenyina

A greater development of the triturating surface of the jaws is characteristic of the *Stereogenyina* among podocnemidids. Similar conditions have been investigated for other turtle groups and related to their feeding habits. Analyses of the skull and lower jaw morphology of bothremydids from the Dakhla Formation, Late Cretaceous of Egypt, suggest that the well-developed maxillary region of those turtles were related to a durophagous diet, composed mainly of hard shell/carapace preys, such as ammonites, oysters, and arthropods (Lapparent de Broin & Werner, 1998; Gaffney, Tong & Meylan, 2006; Rabi, Vremir & Tong, 2013). More recently, Claude et al. (2004) analyzed the skull morphology of 85 species of Testudinoidea using geometric morphometrics, and found that a durophagous diet significantly changes the morphology of the rostral part of the skull, independently of their phylogenetic relations. Species with this feeding habit are “characterized by a wide expansion of the triturating surface and secondary palate” (Claude et al., 2004).

Aiming at exploring this correlation for *Stereogenyina* turtles, we conducted two geometric morphometric analyses, for the upper and lower jaws, including, respectively, 14 and 15 living taxa with known feeding habits (Moll , 1976; Meylan, 1988; Burke, Morreale & Standora, 1994; Teran, Vogt & Gomez, 1995; Pérez-Emán & Paolillo, 1997; Claude et al., 2004; Polovina et al., 2004; Salmon, Jones & Horch, 2004; Garcia & Lourenco, 2007), plus three extinct *Stereogenyina*. Our results agreed with those found by Claude et al. (2004), suggesting that the triturating surfaces of the upper and lower jaws of durophagous turtles are significantly more expanded caudally and medially than those of non-durophagous species (Fig. 7; Table 2). This difference in morphology implies an increase in the area of both the upper and lower jaws used for processing food. At the same time, the morphology of the three included *Stereogenyina* is very similar to that of the durophagous turtles (Figs. 7B and 7C) and significantly different from those of the non-durophagous species (Table 2), supporting the hypothesis that stereogenyins were adapted to a durophagous feeding strategy.

### Palaeohabitat of Stereogenyina

In the original description of *“Podocnemis” venezuelensis*, Wood & Díaz de Gamero (1971) raised the possibility that it could represent a marine turtle. Yet, they at the time asserted that: “to be able to do so (i.e., confirm that *Podocnemis venezuelensis* was a marine turtle) would be particularly interesting because, while all living pelomedusids (*Pelomedusoides*) are inhabitants of fresh waters, in the past some were marine (…). If *P. venezuelensis* were, in fact, marine, it would be the last recorded pelomedusid so adapted”.

More recently, in the systematics and morphology review of *Podocnemididae* turtles by Gaffney et al. (2011), the authors also suggested that “many or all (the taxa) of the subtribe Stereogenyina were marine or near-shore marine”. Indeed, the shell morphology of the Urumaco Formation eggs assigned to *B. venezuelensis* by Winkler & Sánchez-Villagra (2006), which differ from that of other podocnemidids, as well as the depositional environment where they were found, indicate that they were deposited in a beach facing a saline body of water, possibly open-sea (Winkler & Sánchez-Villagra, 2006). This led the authors to suggest that at least some species of *Bairdemys* were colonial nesters, laying eggs in beaches and having a marine lifestyle.

The limb morphology of stereogenyin turtles also suggests a similar lifestyle. In the description of *“B.” healeyorum*, Weems & Knight (2013) analysed the morphology of the proximal limb bones, specially the humerus. They found that its elongated head, with the long axis almost parallel to the shaft axis, restricted the movements of the fore limb to the vertical plan. Turtles with this kind of humeral head, e.g., extant marine cheloniid sea turtles, usually have strong aquatic adaptations, with limbs functioning as paddles. These are very capable swimmers, living in large water bodies; and this can be also inferred for stereogenyins.

The paleoenvironments inferred for the deposits where stereogenyins are found also suggest marine habitats. With the exception of *Brontochelys gaffneyi*, the type-locality of which is unknown (Wood, 1970; Gaffney et al., 2011), and *Shweboemys pilgrimi* from the Irrawaddy Beds, in Burma, which is considered of fluvial origin (Thein, Nu & Pyone, 2012), all other *Stereogenyina* come from marine coastal or open sea depositional environments. The Qasr el Sagha Formation is interpreted to include “nearshore marine and alluvial deposits” (Holroyd et al., 1996), and the horizons where *Cordichelys antiqua* and *Stereogenys cromeri* were found also bear shark and sea cow fossils (Andrews, 1906). The Moghra Formation, where *Lemurchelys diasphax* and possibly *Latentemys plowdeni* were found (Gaffney et al., 2011), is interpreted as an estuarine deposit (Hassan et al., 2012) and the Urumaco Formation, where *B. venezuelensis*, *B. sanchezi*, and *B. winklerae* were collected, is currently considered a succession of marine paleoenvironments, maybe related to a lagoon or bay (Smith et al., 2010). The deposits of the Cibao Formation, where *B. hartsteini* was found, are interpreted as a sequence of different marine environments, with different sea levels, including open sea and coastal paleoenvironments (MacPhee & Wyss, 1990). The Chandler Bridge Formation, from North Carolina, USA, where *“B.” healeyorum* was found, is also interpreted as a succession of marine deposits (Sanders, Weems & Lemon, 1982), with little fluvial input (Weems & Knight, 2013). It is dominated by a marine fauna, with sharks, cetaceans, and cheloniid sea turtles, suggesting an open sea environment (Cicimurri & Knight, 2009). Additionally, the Cerro La Cruz deposits, Castillo Formation, which yielded *Bairdemys aff.* MBLUZ-P-5045 (Sánchez-Villagra et al., 2004), also correspond to mainly near-shore marine environments (Wheeler, 1960; Rincón et al., 2014). These are mostly coastal marine records, whereas the Capadare Formation, which yielded the new *Bairdemys thalassica*, was deposited in entirely open sea conditions, with no influence from coastal or continental environments (Lorente, 1978; Díaz de Gamero, 1985). Thus, until now, *B. thalassica* provides the strongest evidence for the marine habits of *Stereogenyina*.

### Salt glands in pleurodiran turtles?

Turtles that invaded saltwater environments, e.g., cheloniid sea turtles (*sensu* Joyce, Parham & Gauthier, 2004), possess orbital salt glands, which function as an osmoregulatory organ, helping their kidneys to excrete the excess of salt (Schmidt-Nielsen & Fange, 1958). Similar glands are also found in all other reptile lineages adapted to marine environments, e.g., birds, crocodiles, and snakes, having independent evolutionary origins (Schmidt-Nielsen & Fange, 1958; Babonis & Brischoux, 2012). These multiple origins are supported by the uneven phylogenetic distribution of the character, as well as by its various anatomical positions. In living turtles, however, the salt gland is always placed behind the orbits (Baccari, Matteo & Minucci, 1992; Babonis & Brischoux, 2012).

The presence of salt glands has been inferred for some extinct reptile. Mesosaurs have a unique foramen nariale obturatum, located behind the external nares, that has been interpreted as an osteological correlate of a salt gland duct (Huene, 1941; Piñeiro et al., 2012). In metriorhynchids, a crocodylomorph lineage highly adapted to marine lifestyle, the presence of salt glands was inferred based on lobulated structures rostral to the orbits (Fernández & Gasparini, 2000; Fernández & Gasparini, 2008; Gandola et al., 2006; Fernández & Herrera, 2009). In *Santanachelys gaffneyi* (Hirayama, 1998), an early cheloniid sea turtle, and the Jurassic plesiochelyids, an extinct lineage of marine turtles, the enlarged foramen interorbitale is used to infer the presence of salt glands (Hirayama, 1998). In all this cases, besides the characteristic morphology of the structures, their anatomical position is also used to suggest the relation to salt glands.

**Figure 9:**
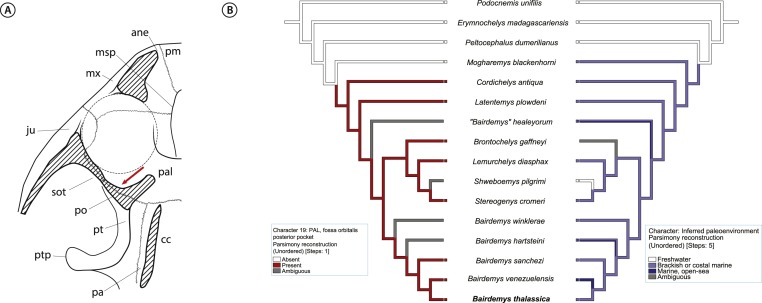
Inference for salt glands in *Stereogenyina* turtles. (A) cross section of a generalized *Stereogenyina* skull, indicating (red arrow) the position of the “posterior pocket on septum orbitotemporale”; (B) distribution of character 19 “fossa orbitalis caudal pocket” (left tree) and inferred paleoenvironment (right tree) in the phylogenetic hypothesis presented here. The coding of the character 19 is based on personal observations and on Gaffney et al. (2011) coding. **Abbreviations: ane,** apertura narium** externa; **cc,** cavum cranii; **ju,** jugal; **msp,** midline cleft of the secondary palate; **mx,** maxilla; **pa,** parietal; **pal,** palatine; **pm,** premaxilla; **po,** postorbital; **pt,** pterygoid; **ptp**, processus trochlearis pterygoidei; **sot,** septum orbitotemporale.

Given the compelling evidence that stereogenyin turtles were adapted to life on sea, they might bear osteological correlates of salt glands. A very good candidate is an excavation, identified by Gaffney, Tong & Meylan (2006) (character 27) as synapomorphic for Bothremydidae, and by Gaffney et al. (2011; character 27) as the “fossa orbitalis posterior pocket in septum orbitotemporale”, seen in all *Stereogenyina* in which that area is exposed (Fig. 9A). In fact, the authors speculate that it could contain “eyeball attachments or orbital glands”. Indeed, its location is comparable to that of salt glands in extant turtles (Baccari, Matteo & Minucci, 1992; Babonis & Brischoux, 2012), and the prevalence of such an organ in extant marine reptiles suggests the “fossa orbitalis posterior pocket in septum orbitotemporale” as a possible osteological correlate of salt glands in *Stereogenyina* turtles.

## Conclusions

A turtle skull from the middle Miocene Capadare Formation, Venezuela, is described as the holotype of the new species, *Bairdemys thalassica*. Two autapomorphies were recognized for this new taxon: 1—dermal scale iv expanding onto the postorbital, quadratojugal, and parietal; 2—slitlike opening of the canal containing both foramina nervi hypoglossi significantly smaller than the foramen jugulare posterius. Recovered as the sister-taxon to *Bairdemys venezuelensis*, *B. thalassica* adds new information on the morphology and phylogeny of *Stereogenyina*. The addition of the new taxon and of new characters to a phylogenetic data matrix changed the position of some stereogenyins, e.g., *Cordichelys antiqua*, *Latentemys plowdeni*, and *“Bairdemys” healeyorum*, the latter being recovered outside *Bairdemys*. The new set of relations suggest that some traits were more widely distributed among *Stereogenyina*, including a secondary palate with curved medial edges.

Morphometric analyses revealed that *Stereogenyina* turtles probably had a durophagous diet, and a review of the morphology and geological provenance of *Stereogenyina* indicates that those pelomedusoids had a marine lifestyle. These two features are also seen in the extant Carettini sea turtles, which began their diversification around the middle Miocene (Dodd & Morgan, 1992; Parham & Fastovsky, 1997; Parham & Pyenson, 2010) and may have since competed with the stereogenyins. This is consistent with the decline of the latter group from the late Miocene onwards (Figs. 8B and 8C), which may have been partially driven by the rise of Carettini, although a much better fossil record of both groups is necessary to test that hypothesis.

## Supplemental Information

10.7717/peerj.1063/supp-1 Supplemental Information 1 Data matrix Taxon-character matrix used in the present phylogenetic analysis.

10.7717/peerj.1063/supp-2 Supplemental Information 2 Character names list List of the 57 characters and character state names used in the phylogenetic analysis.

10.7717/peerj.1063/supp-3 Supplemental Information 3 List of employed specimens and description of the landmarks used in the geometric morphometric analyses List of the taxa used in the geometric morphometric analyses including their specific names, relationships, specimen numbers, and diet informations, and description of the landmarks employed in the analyses for the upper and lower jaws.

## Additional Information and Declarations

### Data Deposition

The following information was supplied regarding the deposition of related data: MorphoBank: http://www.morphobank.org/index.php/Projects/ProjectOverview/project_id/2194.

### New Species Registration

The following information was supplied regarding the registration of a newly described species: New taxon LSID: urn:lsid:zoobank.org:act:D2192A69-5019-410A-A875-48097A50F8CD.
