# Supplementary material for: The last marine pelomedusoids (Testudines: Pleurodira): a new species of Bairdemys and the paleoecology of Stereogenyina
Source: PeerJ. 2015 Jun 30;3:e1063. doi: 10.7717/peerj.1063 (PMC4493680; doi:10.7717/peerj.1063)
Supplement: Supplemental Information 1 — Taxon-character matrix used in the present phylogenetic analysis. [file peerj-03-1063-s001.docx]

## The Last Marine Pelomedusoids: a new species of *Bairdemys* and the Paleoecology of *Stereogenyina*

Gabriel S. Ferreira^1^, Ascanio D. Rincón^2^, Andrés Solórzano^2^, Max C. Langer^1^

^1^Laboratório de Paleontologia de Ribeirão Preto, FFCLRP, Universidade de São Paulo, Avenida Bandeirantes 3900, 14040-901, Ribeirão Preto, SP, Brazil

^2^Laboratorio de Paleontología, Centro de Ecología, Instituto Venezolano de Investigaciones Científcas (IVIC), Carretera Panamericana Km 11, 1020-A, Caracas, Venezuela

**Supplementary File 1 - Data matrix**

*Podocnemis unifilis*

00100000000000000000000-0000001000100000000000000[0 1][0 1]00001[0 1]

*Peltocephalus dumerilianus*

000021?0011?01000000000-000000000000000000000000100011100

*Erymnochelys madagascariensis*

000001?0000001000000000-000000000000000000000000111000011

*Mogharemys* *blackenhorni*

?0?1??0?????110?0?01100-010100?00??1100?00???????????????

*Brontochelys gaffneyi*

10?1??0?????1011011?1110010????????1?00?10???????????????

*Lemurchelys* *diasphax*

10?1??0??111101101111111010?11110??0000110???????????????

*Shweboemys pilgrimi*

10?1??1??111101101?11111211????????101??10???????????????

*Stereogenys cromeri*

10?11?1001??101101111111211111110??1010111111?11?????????

*Cordichelys antiqua*

00111010001?0011011?1111?10100?10?11100110??????211001101

*Latentemys plowdeni*

01?1??0000??0011011?1110010?00011??1100110???????????????

*Bairdemys thalassica*

??1110011???001?1110111?110111111111101111???????????????

*"Bairdemys" healeyorum*

??????????????????????????????111?????????1011??211111101

*Bairdemys venezuelensis*

01111001100?0011111011101101111111111011111111111--11?101

*Bairdemys hartsteini*

01?11?01100?001111?011100?0110111?11100110???????????????

*Bairdemys sanchezi*

0111000110??0011011?11100?0110111?10101111111111?????????

*Bairdemys winklerae*

011??001111?001111??11100????0111111100110???????????????
