# Supplementary material for: The last marine pelomedusoids (Testudines: Pleurodira): a new species of Bairdemys and the paleoecology of Stereogenyina
Source: PeerJ. 2015 Jun 30;3:e1063. doi: 10.7717/peerj.1063 (PMC4493680; doi:10.7717/peerj.1063)
Supplement: Supplemental Information 2 — List of the 57 characters and character state names used in the phylogenetic analysis. [file peerj-03-1063-s002.docx]

## The Last Marine Pelomedusoids: a new species of *Bairdemys* and the Paleoecology of *Stereogenyina*

Gabriel S. Ferreira^1^, Ascanio D. Rincón^2^, Andrés Solórzano^2^, Max C. Langer^1^

^1^Laboratório de Paleontologia de Ribeirão Preto, FFCLRP, Universidade de São Paulo, Avenida Bandeirantes 3900, 14040-901, Ribeirão Preto, SP, Brazil

^2^Laboratorio de Paleontología, Centro de Ecología, Instituto Venezolano de Investigaciones Científcas (IVIC), Carretera Panamericana Km 11, 1020-A, Caracas, Venezuela

**Supplemental File 2 - Character name list**

**Abbreviations: BF05,** Bona & de la Fuente 2005; **C12,** Cadena et al 2012; **D13,** Dumont-Júnior 2013; **F03,** de la Fuente 2003; **G06,** Gaffney, Tong & Meylan 2006; **G11,** Gaffney et al. 2011; **G77,** Gaffney 1977; **M09,** Meylan, Gaffney & Campos 2009; **TG09,** Thomson & Georges 2009

1. PF, preorbital skull broad (G06Ch05, C12Ch08): (0) narrow, as in *Podocnemis unifilis* or (1) very broad, as in *Brontochelys gaffneyi*

2. FR, prefrontal/frontal (G11Ch04): (0) flat or slight convex (*Podocnemis unifilis*) or (1) strongly convex dorsally (*Bairdemys venezuelensis*)

3. Cheek Emargination, extension in lateral view (G11Ch11, BF05Ch01, BF05Ch08, G77Ch10): (0) absent or slight (*Peltocephalus dumerilianus*) or (1) reaches ventral margin level of orbit (*Bairdemys thalassica*) [Ordered]

4. PA, parietal-pterygoid contact in *septum orbitotemporale* (G11Ch06): (0) absent (*Podocnemis unifilis*) or (1) present (*Bairdemys venezuelensis*)

5. PA, χ (interparietal) scale (G11ch08): (0) elongate triangle (*Podocnemis unifilis*) or (1) parallel sided (*Bairdemys thalassica*) or (2) broad posteriorly (*Peltocephalus dumerilianus*)

6. JU, ju-qu contact (G06Ch22, G11Ch09): (0) absent (*Podocnemis unifilis*) or (1) yes (*Peltocephalus dumerilianus*)

7. JU, jugal-pterygoid contact (New character): (0) yes (*Bairdemys venezuelensis*) or (1) no (*Stereogenys cromeri*)

8. SQ, caudal projection (G06Ch24): (0) lacks projection (*Cordichelys antiqua*) or (1) forms distinct process, projecting caudally (*Bairdemys thalassica*)

9. SQ, posteroventral vertical flange (G06Ch25, G11Ch12): (0) absent (*Cordichelys antiqua*) or (1) present (*Bairdemys thalassica*)

10. PM, pinched snout (G11Ch15): (0) absent, convex or straight rostral outline (*Bairdemys venezuelensis*) or (1) present, concave outline near premaxilla-maxilla contact (*Bairdemys winklerae*)

11. PM, cranial pit on ventral surface (New character): (0) no (*Bairdemys venezuelensis*) or (1) yes (*Bairdemys winklerae*)

12. PM, *foramen prepalatinum* relative to triturating ridge (M09Ch15): (0) under triturating surface (*Podocnemis unifilis*) or (1) on flat surface (*Shweboemys pilgrimi*)

13. MX, labial ridge (G11Ch19): (0) high (*Bairdemys venezuelensis*) or (1) low (*Stereogenys cromeri*)

14. MX, lingual ridge (New character): (0) absent or slight (*Bairdemys venezuelensis*) or (1) present (*Peltocephalus dumerilianus*)

15. MX, triturating surfaces (G06Ch34): (0) narrow as in *Podocnemis unifilis* or (1) very wide as in *Stereogenys cromeri*

16. MX, triturating surfaces shape (New character): (0) lingual and labial ridges parallel sided (*Podocnemis unifilis*) or (1) triangular, wide caudally (*Stereogenys cromeri*)

17. MX, triturating surface convexity (G11Ch18): (0) absent or shallow (*Stereogenys cromeri*) or (1) deep (*Bairdemys venezuelensis*)

18. MX, accessory ridge (G06Ch36, G11Ch20): (0) present (*Podocnemis unifilis*) or (1) absent (*Bairdemys venezuelensis*)

19. PAL, *fossa orbitalis* caudal pocket (G06Ch27, G11Ch27): (0) absent (*Podocnemis unifilis*) or (1) present in *septum orbitotemporale* (*Bairdemys venezuelensis*)

20. PAL, *foramen palatinum posterius* (C12Ch27): (0) present (*Podocnemis unifilis*) or (1) absent (*Stereogenys cromeri*) [Ordered]

21. PAL, dorsally arched palate (G06Ch49): (0) absent (*Podocnemis unifilis*) or (1) present (*Bairdemys venezuelensis*)

22. PAL, palatine extent in triturating surface (G06Ch50, G11Ch24): (0) moderate, but much less than extent of maxilla (*Podocnemis unifilis*) or (1) large, equal to or slightly less than extent of maxilla (*Bairdemys venezuelensis*)

23. PAL, secondary palate (C12Ch28, G11Ch23): (0) absent (*Podocnemis unifilis*) or (1) present (*Bairdemys venezuelensis*)

24. PAL, secondary palate, medial edges of palatal cleft (G11Ch17): (0) curved (*Bairdemys venezuelensis*) or (1) parallel (*Stereogenys cromeri*)

25. PAL, caudal development (G11Ch28): (0) poorly developed (*Podocnemis unifilis*) or (1) developed, reducing the contact between pterygoids as in *Baidemys venezuelensis* or (2) well-developed, reaching the basisphenoid as in *Stereogenys cromeri*

26. PAL, dorsal process reaches frontal (G11Ch26): (0) no (*Podocnemis unifilis*) or (1) yes (*Stereogenys cromeri*)

27. PAL, dorsal process of palatine contacts parietal in *septum orbitotemporale* (G11Ch25): (0) no (*Bairdemys venezuelensis*) or (1) yes (*Stereogenys cromeri*)

28. QU, *fossa precolumellaris* (G06Ch56, G11Ch30, C12Ch16): (0) present (*Podocnemis unifilis*) or (1) very small to absent (*Bairdemys thalassica*)

29. QU, entrance of the *antrum postoticum* (G06Ch51, G11Ch29, F03Ch22): (0) small (*Latentemys plowdeni*) or (1) slitlike (*Bairdemys thalassica*)

30. QU, *condylus mandibularis* position (G06Ch60): (0) rostral to bo-bs suture (*Latentemys plowdeni*) or (1) caudal to or on bo-bs suture (*Bairdemys venezuelensis*)

31. QU, *condylus mandibularis* shape (C12Ch29): (0) much wider than long, with anterior and posterior edges straight to concave making it shorter at midline (*Peltocephalus dumerilianus*) or (1) slightly wider than long in a 'kidney bean' shape, with anterior edge straight to concave and posterior edge convex (*Bairdemys thalassica*)

32. QU, ventral outline in lateral view (C12Ch17, F03Ch18): (0) smooth, *condylus mandibularis* very close to the *cavum tympani* region (*Podocnemis unifilis*) or (1) ventral projection, *condylus mandibularis* separated from the *cavum tympani* region (*Bairdemys thalassica*)

33. QU, eustachian tube separated from *fenestra postotica* (G11Ch31): (0) no (*Cordichelys antiqua*) or (1) yes (*Bairdemys thalassica*)

34. SO, bulbous ending of *crista occipitalis* (C12Ch11): (0) flat ending (*Podocnemis unifilis*) or (1) bulbous ending (*Bairdemys thalassica*)

35. SO, horizontal plate along ventral edge of *crista supraoccipitalis* (G11Ch40): (0) flat or very slim horizontal plate on ventral edge (*Peltocephalus dumerilianus*) or (1) horizontal plate along ventral edge (*Bairdemys thalassica*)

36. EX, *foramina nervi hypoglossi* (G11Ch43): (0) separated (*Podocnemis unifilis*) or (1) combined and recessed (*Bairdemys thalassica*)

37. EX, exoccipital-quadrate contact (G06Ch85, C12Ch30): (0) no contact (*Stereogenys cromeri*) or (1) exoccipital contacts quadrate (*Bairdemys venezuelensis*)

38. BO, length (G06Ch87, G11Ch44, C12Ch21): (0) long to short length/width => 0.25 (*Podocnemis unifilis*) or (1) very short, length/width < 0.25 (*Shweboemys pilgrimi*)

39. OP, projects ventrally forming a flange over the *foramen jugulare posterius* (New character): (0) no (*Podocnemis unifilis*) or (1) yes (*Bairdemys sanchezi*)

40. OP, *processus paraoccipitalis* in ventral view (G06Ch102, C12Ch22): (0) projects caudally beyond squamosal (*Podocnemis unifilis*) or (1) smaller, rostral to squamosal (*Bairdemys venezuelensis*)

41. BS, ventral outline (G06Ch106): (0) pentagonal (*Podocnemis unifilis*) or (1) triangular (*Bairdemys venezuelensis*)

42. BS, bs-qu contact (G06Ch104, C12Ch23, F03Ch12, G11Ch53, BF05Ch13): (0) wider contact (*Podocnemis unifilis*) or (1) narrow contact (*Bairdemys venezuelensis*)

43. DEN, lingual ridge (G06Ch114): (0) high (*Podocnemis unifilis*) or (1) low (*Bairdemys venezuelensis*)

44. DEN, symphyseal hook (TG09Ch22): (0) hooked rostral edge (*Peltocephalus dumerilianus*) or (1) no hook on rostral edge (*Bairdemys venezuelensis*)

45. DEN, triturating surfaces (New character): (0) narrow (*Podocnemis unifilis*) or (1) wider (*Bairdemys venezuelensis*)

46. COR, wide lateral exposure (G06Ch121): (0) no (*Podocnemis unifilis*) or (1) yes (*Bairdemys venezuelensis*)

47. COR, participates on triturating surface (New character): (0) yes, lingual-caudal portion formed by coronoid (*Podocnemis unifilis*) or (1) yes, caudal portion formed by coronoid (*Stereogenys cromeri*)

48. ART, *processus retroarticularis* (G06Ch123, G11Ch55): (0) long, caudoventral (*Podocnemis unifilis*) or (1) long, caudal (*Bairdemys venezuelensis*)

49. NU, proportions (New character): (0) anterior width/greatest width < 0.40 (*Podocnemis unifilis*) or (1) 0.70 < anterior width/greatest width >= 0.40 (*Peltocephalus dumerilianus*) or (2) anterior width/greatest width > 0.70 (*Cordichelys antiqua*)

50. CAR, neural number (G06Ch146, G11Ch64, C12Ch42): (0) 7 neurals (*Peltocephalus dumerilianus*) or (1) 6 neurals (*Cordichelys antiqua*) [Ordered]

51. CAR, neural series completeness (G06Ch141, G11Ch63, F03Ch38): (0) to costals 7 (*Peltocephalus dumerilianus*) or (1) to costals 6 (*Cordichelys antiqua*) [Ordered]

52. CAR, intumescence from axillary buttress to the rib area on costal 1 (New character): (0) no (*Podocnemis unifilis*) or (1) yes (*Bairdemys venezuelensis*)

53. CAR, axillary buttress (G06Ch149, G11Ch68, BF05Ch32): (0) reaches peripheral 2 (*Podocnemis unifilis*) or (1) reaches peripheral 3 (*Bairdemys venezuelensis*)

54. CAR, second vertebral scute shape (D13Ch103): (0) hexagonal (*Podocnemis unifilis*) or (1) quadrilateral (*Cordichelys antiqua*)

55. PLA, anterior lobe length (G06Ch164): (0) long to medium, width over length < = 2.0 (*Podocnemis unifilis*) or (1) short, width over length > 2.1 (*Cordichelys antiqua*)

56. PLA, posterior lobe lateral outline (D13Ch188): (0) almost straight lined (*Bairdemys venezuelensis*) or (1) convex (*Podocnemis unifilis*)

57. PLA, pectoral on epiplastron (G06Ch166, G11Ch74, F03Ch36): (0) no, far behind epiplastron (*Peltocephalus dumerilianus*) or (1) on epiplastron, on epi-hyoplastron suture (*Bairdemys venezuelensis*)
