# Supplementary material for: The last marine pelomedusoids (Testudines: Pleurodira): a new species of Bairdemys and the paleoecology of Stereogenyina
Source: PeerJ. 2015 Jun 30;3:e1063. doi: 10.7717/peerj.1063 (PMC4493680; doi:10.7717/peerj.1063)
Supplement: Supplemental Information 3 — List of the taxa used in the geometric morphometric analyses including their specific names, relationships, specimen numbers, and diet informations, and description of the landmarks employed in the analyses for the upper and lower jaws. [file peerj-03-1063-s003.docx]

## The Last Marine Pelomedusoids: a new species of *Bairdemys* and the Paleoecology of *Stereogenyina*

Gabriel S. Ferreira^1^, Ascanio D. Rincón^2^, Andrés Solórzano^2^, Max C. Langer^1^

^1^Laboratório de Paleontologia de Ribeirão Preto, FFCLRP, Universidade de São Paulo, Avenida Bandeirantes 3900, 14040-901, Ribeirão Preto, SP, Brazil

^2^Laboratorio de Paleontología, Centro de Ecología, Instituto Venezolano de Investigaciones Científcas (IVIC), Carretera Panamericana Km 11, 1020-A, Caracas, Venezuela

**Supplemental file 3 - List of employed specimens and description of the landmarks used in the geometric morphometric analyses**

| **Palate Analysis** |  |  |  |
| --- | --- | --- | --- |
| Species | Higher clade | Specimen | Diet |
| *Chelonia mydas* | Chelonidae | AMNH 5912 | Non-durophagous |
| *Chelonoids denticulata* | Testudinidae | USNM 73932 C | Non-durophagous |
| *Dermochelys coriaceae* | Dermochelyidae | AMNH 7160 | Non-durophagous |
| *Eretmochelys imbricata* | Chelonidae | ZMB 46556 | Non-durophagous |
| *Erymnochelys madagascariensis* | Podocnemididae | SMF 7979 | Non-durophagous |
| *Peltocephalus dumerilianus* | Podocnemididae | INPA-H 22885 | Non-durophagous |
| *Podocnemis expansa* | Podocnemididae | MPEG 0292 | Non-durophagous |
| *Podocnemis unifilis* | Podocnemididae | INPA-H 8942 | Non-durophagous |
| *Trachemys scripta* | Emydidae | UCMP 138016 | Non-durophagous |
| *Geoclemys hamiltonii* | Geomydidae | CRI 487 | Durophagous |
| *Graptemys barbouri* | Emydidae | UCMP 130640 | Durophagous |
| *Graptemys pseudogeographica* | Emydidae | USNM 24695 1 | Durophagous |
| *Lepidochelys olivacea* | Chelonidae | ZMB 51999 | Durophagous |
| *Malayemys subtrijuga* | Geomydidae | PCHP 3446 | Durophagous |
| *Bairdemys venezuelensis* | Podocnemididae | MCNC-Pal-21-10708 | Unknown |
| *Bairdemys winklerae* | Podocnemididae | AMU-CURS-98 | Unknown |
| *Lemurchelys diasphax* | Podocnemididae | DPC 6425 | Unknown |
|  |  |  |  |
| **Lower Jaw Analysis** |  |  |  |
| Species | Higher clade | Specimen | Diet |
| *Chelonia mydas* | Chelonidae | AMNH 5912 | Non-durophagous |
| *Chelonoids denticulata* | Testudinidae | USNM 73932 M | Non-durophagous |
| *Dermochelys coriaceae* | Dermochelyidae | MNHN Pal 1870-101 | Non-durophagous |
| *Eretmochelys imbricata* | Chelonidae | ZMB 46556 | Non-durophagous |
| *Erymnochelys madagascariensis* | Podocnemididae | SMF 7979 | Non-durophagous |
| *Peltocephalus dumerilianus* | Podocnemididae | INPA-H 22885 | Non-durophagous |
| *Podocnemis expansa* | Podocnemididae | MPEG 0292 | Non-durophagous |
| *Podocnemis unifilis* | Podocnemididae | INPA-H 8942 | Non-durophagous |
| *Trachemys scripta* | Emydidae | UCMP 138016 2 | Non-durophagous |
| *Caretta caretta* | Chelonidae | ZMB 46511 | Durophagous |
| *Geoclemys hamiltonii* | Geomydidae | CRI 487 | Durophagous |
| *Graptemys geographica* | Emydidae | UCMVZ 222414 2 | Durophagous |
| *Graptemys pseudogeographica* | Emydidae | USNM 24695 2 | Durophagous |
| *Lepidochelys olivacea* | Chelonidae | ZMB 51999 | Durophagous |
| *Malayemys subtrijuga* | Geomydidae | PCHP 3446 | Durophagous |
| *Bairdemys healeyorum* | Podocnemididae | SC 90-16 | Unknown |
| *Bairdemys venezuelensis* | Podocnemididae | MCNC Pal-26A-72V | Unknown |
| *Stereogenys cromeri* | Podocnemididae | UM 161 | Unknown |

**Abbreviations: AMNH:** American Museum of Natural History, New York, USA; **AMU-CURS:** Alcadía del Municipio de Urumaco, Colección Rodolfo Sánchez, Urumaco, Venezuela; **CRI:** collection of the Chelonian Research Institute, Oviedo, USA ; **DPC:** Duke Primate Center, Durham, USA; **INPA-H**: Coleção de Herpetologia do Instituto Nacional de Pesquisas da Amazonia, Manaus, Brazil; **MCNC-PAL:** Colección de Paleontología del Museu de Ciencias Naturales de Caracas, Caracas, Venezuela; **MNHN:** Muséum National d'Histoire Naturelle, Paris, France; **MPEG:** Museu Paraense Emilio Goeldi, Belém, Brazil; **PCHP:** collection of the Chelonian Research Institute, Oviedo, USA; **SC:** South Carolina State Museum, Columbia, USA; **UCMP:** University of California Museum of Paleontology, Berkeley, USA; **UCMVZ:** ; **UM:** Montpellier University, Montpellier, France; **USNM:** United States National Museum, Washington, DC, USA; **ZMB:** Museum für Naturkunde, Berlin, Germany;

**Description of the landmarks in the upper jaw**

| Landmark | Description |
| --- | --- |
| 1 | Rostral edge of the triturating surface on the contact between the premaxillae |
| 2 | Contact between the left premaxilla and maxilla on the labial ridge of the triturating surface |
| 3 | Median distance between landmarks 1 and 4 |
| 4 | Left caudolateral edge of the triturating surface |
| 5 | Left caudomedial edge of the triturating surface |
| 6 | Median distance between landmarks 5 and 7 |
| 7 | Caudal edge of the midline extension of the triturating surface |
| 8 | Median distance between landmarks 7 and 9 |
| 9 | Right caudomedial edge of the triturating surface |
| 10 | Right caudolateral edge of the triturating surface |
| 11 | Median distance between landmarks 10 and 1 |
| 12 | Contact between the right premaxilla and maxilla on the labial ridge of the triturating surface |

Description of the landmarks in the lower jaw

| Landmark | Description |
| --- | --- |
| 1 | Rostral edge of the triturating surface |
| 2 | Median distance between landmarks 1 and 3 |
| 3 | Left caudolateral edge of the triturating surface |
| 4 | Left caudomedial edge of the triturating surface |
| 5 | Median distance between landmarks 4 and 6 |
| 6 | Caudal edge of the midline extension of the triturating surface |
| 7 | Median distance between landmarks 6 and 8 |
| 8 | Right caudomedial edge of the triturating surface |
| 9 | Right caudolateral edge of the triturating surface |
| 10 | Median distance between landmarks 9 and 1 |
